# On Definitions of Constants and Types in HOL

**DOI:** 10.1007/s10817-016-9366-4

**Published:** 2016-03-08

**Authors:** Rob Arthan

**Affiliations:** 1 Lemma 1 Ltd., Reading, UK; 2 Department of Computer Science, University of Oxford, Oxford, UK

**Keywords:** Higher-order logic, Interactive theorem proving, Conservative extension

## Abstract

This paper reports on a simpler and more powerful replacement for the principles for defining new constants that were previously provided in the various HOL implementations. We discuss the problems that the new principle is intended to solve and sketch the proofs that it is conservative and that it subsumes the earlier definitional principles. The new definitional principle for constants has been implemented in HOL4 and in ProofPower and has been adopted in OpenTheory and in the work of Kumar, Myreen and Owens on a fully verified implementation of HOL. Kumar et al. have formally verified that the new definitional principle is conservative with respect to the standard set theoretic semantics of HOL. We continue this line of thought with a look at the mechanisms for defining new types and consider potential improvements, one of which has now been adopted in OpenTheory.

## Introduction

Pitching specifications at an appropriate degree of precision and generality is an important aspect of systems engineering. In mathematics, it is vital to choose an appropriate set of abstract concepts on which to found a theory. Nonetheless, in the automated reasoning community we often seem to underplay the importance of clear, abstract definitions, with many developments being founded on rather crude axiomatisations. The widely used HOL logic contains some features intended to support abstract specifications. This paper discusses these features and some potential improvements to them.

The design of the HOL logic and of its definitional principles [2] evolved in the late 80s and early 90s. Some form of this design has been implemented in HOL4 [16, 17], HOL Light [8], HOL Zero [1], Isabelle/HOL [15], OpenTheory [11] and ProofPower [4]. While the definitional principles have stood the test of time in many practical applications, we believe there is still some room for improvement. We will discuss issues with the mechanisms for introducing new constants and types and consider new and more general mechanisms to address these issues.

The discussion of constant definitions in this paper is based on work originally presented at ITP2014 [3]. The discussion of type definitions is new for this special issue of the Journal of Automated Reasoning.

## Definitional Principles in Logic

We begin by defining some terminology for use in discussing definitional principles in logic. This is largely technical and the reader may wish to skip it on a first reading referring back to it as necessary when later on we talk about consistency or conservativeness.

To discuss the notion of a definitional principle, we assume given a language equipped with an inference system or a semantics. We assume the language is parametrised by a signature defining a set of primitive constructs. In the sequel, we will solely be concerned with the HOL language and inference system as defined in [8]. Signatures will be as in [2] and comprise sets of type constructor names and associated arities and sets of constant names and associated types. The inference system of [8] can be shown to be equivalent to the somewhat different system used in HOL4 and ProofPower as defined in [2]. The judgments of the inference system are sequents $t_1, \ldots , t_n \vdash t$, where *t* and the $t_i$ are terms of type ${\mathsf {bool}}$.

A *definitional principle* is a generalised inference rule that includes a signature extension. An instance of such a principle has antecedents comprising a (possibly empty) list of judgments of some prescribed form over some signature $\varSigma$ and has succedents comprising a list of judgments over some extended signature $\varSigma '$. Intuitively, the succedents are axioms that specify the intended meaning of the new primitive constructs introduced in $\varSigma '$, while the antecedents provide evidence that these axioms are consistent (or, even better, conservative, as discussed below). We define a context $\varGamma = (\varSigma , A)$ to be a pair comprising a signature and a set of axioms. Definitional principles are applied sequentially to obtain new contexts from old ones starting from some initial context $\varGamma _0$. It is only valid to apply a definitional principle to a context $\varGamma = (\varSigma , A)$ if its antecedents are in the language over $\varSigma$ and are derivable from the axioms *A*. The resulting context $\varGamma '$ is $(\varSigma ', A \cup S)$ where $\varSigma '$ is the extended signature and *S* is the set of succedent theorems associated with this instance of the definitional principle.

In later sections, we will usually think of a definitional principle operationally and hence refer to it as taking some inputs and introducing new primitives and associated axioms. In practice, an implementation will often require input parameters in addition to the antecedent theorems in order to describe the form of the signature extension, e.g., to give the name of a new constant.

We say that a definitional principle is *consistent* if, whenever it is validly applied to a context $\varGamma = (\varSigma , A)$ to give a new context $\varGamma ' = (\varSigma ', A')$, then $A'$ is consistent if *A* is consistent.

We say that a definitional principle is *model-theoretically conservative* with respect to a semantics, if, whenever it is validly applied to a context $\varGamma$ to give a new context $\varGamma '$, then any model of $\varGamma$ may be expanded to a model of $\varGamma '$.

We say that a definitional principle is *proof-theoretically conservative* if, whenever it is validly applied to a context $\varGamma = (\varSigma , A)$ to give a new context $\varGamma ' = (\varSigma ', A')$, then any judgment over $\varSigma$ that is derivable from $A'$ is also derivable from *A*.

Conservativeness, in either the model-theoretic or proof-theoretic sense, implies consistency. In general, consistency is a much weaker property than either kind of conservativeness. The two notions of conservativeness coincide for a logic that is sound and complete for the semantics in question. However, HOL is sound but not complete for its standard semantics. In the sequel when we refer to model-theoretic notions in HOL, we will always mean the standard semantics unless otherwise stated. See [2] for a readable and rigorous account of the standard semantics for HOL. Note that the standard semantics actually defines a proper class of standard models: “standard” means that function types are modelled by function spaces in the meta-logic and does not imply any restriction on the cardinality of a model (hence the careful use of the phrase “a standard model” in [2]).

## On Defining Constants

### The Existing Mechanisms

The original Classic HOL provided a mechanism for defining new constants known as new_definition. This worked as follows: given a possibly empty list of variables $x_1,\ldots ,x_n$ and a term *t* whose free variables are contained in the $x_i$, it introduced a new constant^1^ *c* of the appropriate type and the axiom:

$$\begin{aligned} \vdash \forall x_1\,\ldots \,x_n {\cdot }\, c\;x_1\;\ldots \;x_n = t. \end{aligned}$$

This simple mechanism is remarkably powerful but suffered from two significant shortcomings, both pointed out by Roger Jones^2^:

**RJ1**: The mechanism does not support implicit definitions. As one example, it is pleasant to define the destructors of a data type as the left inverses of the constructors. Thus one wants to define ${\mathsf {Pre}}$ in terms of ${\mathsf {Suc}}$ by:
  $$\begin{aligned} {\mathsf {Pre}}({\mathsf {Suc}}(n)) = n. \end{aligned}$$
  As another example, the exponential function is naturally defined by a differential equation:
  $$\begin{aligned} {\mathsf {exp}}(0)= & {} 1 \\ ({\mathsf {D}}\,{\mathsf {exp}})(x)= & {} {\mathsf {exp}}(x). \end{aligned}$$
  In such cases, the mechanism can be used to define constants having the desired properties, but one has to use the Hilbert choice operator to give witnesses and then derive the implicit definitions as theorems. This results in a loss of abstraction and unintended identities, e.g., the naive way of defining two constants $c_1$ and $c_2$ both with the loose defining property $c_i \le 10$ will result in an extension in which $c_1 = c_2$ is provable.
**RJ2**: The mechanism is unsound. The condition on the free variables of *t* is certainly necessary. Without it, we could take *t* to be a variable, $y:\mathbb {N}$, and define a new constant *c* satisfying $\vdash \forall y : \mathbb {N} {\cdot }\, c = y.$ Specialising this in two different ways, we could prove both $c = 1$ and $c = 2$. However, the condition is not sufficient. If $\#$ is a polymorphic function such that $\#X$ is the size of *X* when *X* is a finite set, then we can use the mechanism to define a constant $c:\mathbb {N}$ satisfying the axiom $c = \#\{x:\alpha \mathrel {|}x = x\}$, where $\alpha$ is a type variable. But then if $\mathbf {1}$ and $\mathbf {2}$ denote types with 1 and 2 members respectively, we can instantiate $\alpha$ to prove both $c = \#\{x:\mathbf {1}\mathrel {|}x = x\} = 1$ and $c = \#\{x:\mathbf {2}\mathrel {|}x = x\} = 2$.

The fix for **RJ2** was to change new_definition so as to check that all type variables appearing anywhere in the term *t* also appear in the type of the constant *c* that is being defined. HOL Light, HOL Zero, Isabelle/HOL and ProofPower were all implemented after the problem was known, so they incorporated this solution from scratch. The fix in Classic HOL was carried forward into HOL4.

A new definitional principle was introduced to address **RJ1**. This is called new_specification. It takes as input a theorem of the form $\vdash \exists v_1\,\ldots \,v_n {\cdot }\, p$ and introduces a list of new constants $c_1, \ldots , c_n$ and the axiom

$$\begin{aligned} \vdash p[c_1/v_1, \ldots , c_n/v_n]. \end{aligned}$$

new_specification requires that the free variables of *p* be contained in the $v_i$ and that every type variable appearing anywhere in *p* also appear in the type of each new constant $c_i$, thus avoiding reintroducing the problem of **RJ2** under a different guise. The result is both proof-theoretically and model-theoretically conservative. It also supports a very useful range of implicit definitions. However, there are two issues that I noted during the ProofPower implementation:

**RA1**: Given new_specification, new_definition is redundant: what it does can easily be realised by a derived mechanism that given the list of variables $x_1, \ldots , x_n$ and the term *t*, automatically proves:
  $$\begin{aligned} \vdash \exists y {\cdot }\, \forall x_1\,\ldots \,x_n {\cdot }\, y\;x_1\;\ldots \;x_n = t \end{aligned}$$
  and then applies new_specification. Unfortunately, in order to prove existentially quantified statements, one needs a definition of the existential quantifier, and so new_definition seems necessary to avoid a bootstrapping problem^3^. (Since it is only required for bootstrapping, the ProofPower implementation of new_definition only covers the simple case where the axiom has the form $\vdash c = t$.)
**RA2**: The condition on type variables imposed by new_specification is stronger than one would like. It is natural for certain “concrete” structures to be characterized by more “abstract” properties such as universal mapping properties. For example, data types can be characterized as initial algebras:
  $$\begin{aligned} \forall (z:\alpha ) (r : \alpha \rightarrow \alpha ) {\cdot }\, \exists ! f : \mathbb {N}\rightarrow \alpha {\cdot }\, f(0) = z \wedge \forall n {\cdot }\, f({\mathsf {Suc}}(n)) = r(f(n)). \end{aligned}$$
  However, the above characterization cannot be used as a defining property for the successor function with new_specification. Characterizing objects by universal properties is endemic in modern mathematics and computer science, so it is irritating to be compelled to resort to circumlocutions.

In HOL4, ProofPower and HOL Zero, new_specification is implemented as a primitive operation. However, in HOL Light, it is derived. I believe this was primarily a consequence of the following design goal for HOL Light:

**JH1**: The primitive inference system for HOL Light should be defined in terms of language primitives and equality alone and should not depend on the axiomatization of the logical connectives.

A form of new_specification that does not involve existential quantification was implemented in early versions of HOL Light. This took as input a theorem of the form $\vdash p\;t$. Later, to simplify the correctness argument for the system, new_specification was re-implemented as a derived operation that uses the Hilbert choice operator to translate its inputs into a form suitable for new_definition, applies new_definition, then derives the desired axiom to be passed back to the user from the stronger axiom returned by new_definition. Thus HOL Light bypasses **RA1**, but at the price of a certain inelegance, since we have to trust the derived rule to discard the axiom returned by new_definition. This became worse when HOL Light was enhanced to address the following observation of Mark Adams:

**MA1**: If an LCF style system does not record all the axioms and definitions that have been introduced, the correctness claim for the system has to be defined in terms of a state **and** the sequence of operations which produced that state. This makes it impossible to implement a proof auditing procedure that works by analysing the current state of the system.

As a result of **MA1**, axioms and definitions in HOL Light are now recorded. The current HOL Light implementation uses a trick to prevent two constants with the same loose defining property being provably equal. The trick is based on the following idea: to define $c_1$ and $c_2$ such that $c_1, c_2 \le 10$, say, define $c_1 = (\varepsilon f {\cdot }\, \forall n {\cdot }\, f(n) \le 10)\;1$ and $c_2 = (\varepsilon f {\cdot }\, \forall n {\cdot }\, f(n) \le 10)\;2$; then $c_1$ and $c_2$ have the desired property, but $c_1 = c_2$ is not provable. Nonetheless some unintended identities are still provable that would not be provable if new_specification were implemented as a primitive as in HOL4 or ProofPower.

The equivalent of new_specification in Isabelle/HOL is its **specification** command. This is implemented using an equational definition and the choice function, but that definition only exists in a private namespace. Some aspects of the abstraction offered by new_specification are provided by the very popular locale mechanism in Isabelle.

Quantification over type variables as implemented in HOL-Omega [10] obviates many of the problems discussed here. However, our present concern is with improvements that preserve the delightful simplicity of the Classic HOL logic.

### Proposed Alternative

The proposed alternative is to discard new_definition and to adapt and generalise new_specification so that it does not depend on the meaning of the existential quantifier. We call the generalised new_specification gen_new_specification. It takes as input a theorem of the following form

$$\begin{aligned} v_1 = t_1, \ldots , v_n = t_n \vdash p \end{aligned}$$

where the $v_i$ are variables. If all is well, gen_new_specification will introduce new constants $c_1, \ldots , c_n$ and the following axiom:

$$\begin{aligned} \vdash p[c_1/v_1, \ldots , c_n/v_n]. \end{aligned}$$

gen_new_specification imposes the following restrictions:

- the $v_i$ must be pairwise distinct;
- the terms $t_i$ must have no free variables;
- the free variables of *p* must be contained in the $v_i$;
- any type variable occurring in the type of any subterm of a $t_i$ must occur in the type of the corresponding $v_i$.

There is no restriction on the type variables appearing in *p*.

#### **Claim 1**

gen_new_specification is a conservative definitional principle in both the proof-theoretic and model-theoretic senses.

#### *Proof*

For proof-theoretical conservativeness, assume that a sequent $\varGamma \vdash q$ containing no instances of the $c_i$ is provable using the axiom $\vdash p[c_1/v_1, \ldots , c_n/v_n]$ introduced using gen_new_specification. We will show how to transform a proof tree with conclusion $\varGamma \vdash q$ into a proof tree with the same conclusion that does not use the new axiom. First, by simple equality reasoning, derive from the theorem $v_1 = t_1, \ldots , v_n = t_n \vdash p$ that was passed to new_specification, the theorem $\vdash p[t_1/v_1, \ldots , t_n/v_n].$

Now replace each type instance of a $c_i$ in the proof tree with the corresponding type instance of $t_i$ and wherever a type instance of the axiom $\vdash p[c_1/v_1, \ldots , c_n/v_n]$ is used in the proof tree, replace it with the corresponding type instance of a proof tree for $\vdash p[t_1/v_1, \ldots , t_n/v_n]$. By inspection of the primitive inference rules in [8], if one replaces instances of constants in a correct inference by closed terms of the same type in such a way that assumptions or conclusions of the sequents involved that were syntactically identical before the replacement remain syntactically identical, then the result is also a correct inference. As the condition on type variables imposed by gen_new_specification guarantees that two instances of a $c_i$ are syntactically identical iff the corresponding instances of $t_i$ are syntactically identical, we have constructed a correct proof tree whose conclusion is $\varGamma \vdash q$. That concludes the proof of proof-theoretical conservativeness.

For model-theoretic conservativeness, note that $\exists v_1\,\ldots \,v_n {\cdot }\, p$ is provable using the new axiom by taking the $c_i$ as witnesses, hence by proof-theoretic conservativeness $\exists v_1\,\ldots \,v_n {\cdot }\, p$ is provable without using the new axiom and hence is true in any standard model. Therefore in any standard model, there exist $v_1, \ldots , v_n$ satisfying *p* and these elements may be used to expand the model to a model of the new axiom. $\square$

#### **Claim 2**

gen_new_specification subsumes new_definition.

#### *Proof*

In the simplest case, to define *c* with axiom $\vdash c = t$, where *t* has no free variables and contains no type variables that do not appear in its type, apply gen_new_specification to the axiom $v = t \vdash v = t$. This is all we need to define the logical connectives [8].

For the general case, to define *c* with axiom $\vdash \forall x_1\,\ldots \,x_n {\cdot }\, c\;x_1\ldots \;x_n = t,$

take the axiom $v = (\lambda x_1\,\ldots \,x_n {\cdot }\, t) \vdash v = (\lambda x_1\,\ldots \,x_n {\cdot }\, t)$, derive $v = (\lambda x_1\,\ldots \,x_n {\cdot }\, t) \vdash \forall x_1\,\ldots \,x_n {\cdot }\, v\;x_1\ldots \;x_n = t$ from it and then apply gen_new_specification. $\square$

#### **Claim 3**

gen_new_specification subsumes new_specification.

#### *Proof*

Given the theorem $\vdash \exists v_1\,\ldots \,v_n {\cdot }\,p$, we can derive from it the theorem $v_1 = \varepsilon v_1 {\cdot }\, \exists v_2\,\ldots \,v_n {\cdot }\,p \vdash \exists v_2\,\ldots \,v_n {\cdot }\, p$ and apply gen_new_specification to define a constant $c_1$ with defining axiom $\vdash \exists v_2\,\ldots \,v_n {\cdot }\,p[c_1/v_1]$. Iterating this process we can define $c_2, \ldots , c_n$ such that the defining axiom of $c_n$ is $\vdash p[c_1/v_1, \ldots , c_n/v_n]$. Thus we can achieve the same effect as $\mathtt{new\_specification}$ at the expense of additional intermediate definitions. This is sufficient to define the constructor and destructors for binary products.

Once we have binary products, we can simulate *n*-tuples by iterated pairing. This means that given the theorem $\vdash \exists v_1\,\ldots \,v_n {\cdot }\, p$, we can derive the theorem $\vdash \exists z {\cdot }\, p[\pi _1(z)/v_1, \ldots , \pi _n(z)/v_n]$

in which the *n* bound variables $v_1, \ldots , v_n$ have been collected into a single *n*-tuple denoted by the fresh variable *z* (here $\pi _i$ denotes the projection onto the *i*-th factor). Now we can derive from that the theorem $v_1 = t_1, \ldots , v_n = t_n \vdash p$

where $t_i$ is $\pi _i(\varepsilon z {\cdot }\, p[\pi _1(z)/v_1, \ldots , \pi _n(z)/v_n])$. Given this theorem as input, gen_new_specification has exactly the same effect as new_specification given the input theorem $\vdash \exists v_1 \ldots , v_n {\cdot }\, p$. $\square$

### Assessment

Let me assess the proposed new definitional mechanism gen_new_specification against the observations that led to it:

**RJ1**: By claim 3, the support for implicit definitions is at least as good with gen_new_specification as with new_specification. In fact it is better: using gen_new_specification one can define new constants $f : \alpha \rightarrow \mathbb {N}$ and $n : \mathbb {N}$ with defining property $\forall x {\cdot }\, \lnot f\,x = n$, but this is impossible using new_specification.
**RJ2**: By claim 1, the proposed alternative is sound. What is more, this proof has been formalised in HOL4: Ramana Kumar, Scott Owens and Magnus Myreen have recently completed a formal proof of soundness for the HOL logic and its definitional principles including gen_new_specification [14].
**RA1**: By claim 2, new_definition is no longer required. (The definitions of the connectives as given in [8] only require the simple case in the proof of that claim, so no reasoning about the connectives is needed to define them and there is no bootstrapping issue.)
**RA2**: The restriction on type variables now applies only to the equations that give the witnesses to the consistency of the definition. Defining properties such as initial algebra conditions are supported.
**JH1**: gen_new_specification is defined solely in terms of equality and primitive language constructs.
**MA1**: The unintended identities arising as a result of recording definitions in HOL Light will not occur if gen_new_specification is adopted as the primitive mechanism for defining constants.

gen_new_specification has now been implemented in HOL4 and ProofPower. In both cases it is a replacement for new_definition: the existing new_specification has been retained for pragmatic reasons^4^. The ProofPower implementation includes an implementation of the proof of claim 3 above and this completely replaces new_specification in the development of many of the theories supplied with the system, including all the “pervasive” theories such as the theories of pairs and natural numbers that form part of the logical kernel. gen_new_specification is included in version 6 of OpenTheory as the defineConstList command and is supported by the opentheory tool.

## On Defining Types

For constant definitions, we have offered in section 3 a definite proposal that has been formally verified and adopted in several systems. For type definitions, we feel that there is still work to be done, both on the theory and how to implement it. Nonetheless, we believe that there are definitely some worthwhile alternatives to the existing mechanisms to be considered. In this section we discuss the existing mechanisms and discuss some possible alternatives.

Classic HOL provided a mechanism called new_type_definition for introducing new types that was carried over essentially unchanged into HOL4 and ProofPower. new_type_definition is given a theorem of the form $\vdash \exists x:\sigma {\cdot }\,p\,x$ where *p* is a closed term of type $\sigma \rightarrow {\mathsf {bool}}$. The type variables in *p* (which include those in $\sigma$) must be contained in a given list of type variables $\alpha _1, \ldots , \alpha _n$. new_type_definition introduces a new *n*-ary type constructor, ${\mathsf {op}}$, say^5^ together with the axiom:

$$\begin{aligned} \vdash \exists rep : (\alpha _1, \ldots , \alpha _n){\mathsf {op}}\rightarrow \sigma {\cdot }\, {\mathsf {Type\_Definition}}\,p\, rep \end{aligned}$$

The polymorphic constant ${\mathsf {Type\_Definition}}$ used above is pre-defined with type $(\beta \rightarrow {\mathsf {bool}}) \rightarrow (\alpha \rightarrow \beta ) \rightarrow {\mathsf {bool}}$ and defining property:

$$\begin{aligned} \begin{array}{lll} \vdash {\mathsf {Type\_Definition}}&{}=&{} {} \lambda p\, rep {\cdot }\, (\forall x\,x' {\cdot }\, rep \,x = rep \,x' \Rightarrow x = x') \\ &{}&{}\wedge (\forall y {\cdot }\, p\,y \Leftrightarrow \exists x {\cdot }\, rep \,x = y) \end{array} \end{aligned}$$

So $\exists rep {\cdot }\,{\mathsf {Type\_Definition}}\,p\, rep$ asserts that there exists a one-to-one correspondence between the new type $(\alpha _1, \ldots , \alpha _n){\mathsf {op}}$ and the subset of the existing type $\sigma$ defined by the predicate *p*.

The very simple subtyping mechanism offered by new_type_definition turns out to be very powerful. Let us look in some detail at one of its most fundamental applications, namely the definition of the type of natural numbers. The starting point for this is the type ${\mathsf {ind}}$ of individuals which is axiomatized by the assertion $\exists f : {\mathsf {ind}}\rightarrow {\mathsf {ind}} {\cdot }\, {\mathsf {OneOne}}\,f \wedge \lnot {\mathsf {Onto}}\,f$. Using this and $\mathtt{new\_specification}$, one introduces a predicate ${\mathsf {Is\_}\mathbb {N}\mathsf {\_Rep}}: {\mathsf {ind}}\rightarrow {\mathsf {bool}}$ with defining property:

$$\begin{aligned} \begin{array}{lrl} &{}&{}\vdash \exists z {\cdot }\,\exists s {\cdot }\, {\mathsf {Is\_}\mathbb {N}\mathsf {\_Rep}}\,z \wedge (\forall m {\cdot }\, {\mathsf {Is\_}\mathbb {N}\mathsf {\_Rep}}\,m \Rightarrow {\mathsf {Is\_}\mathbb {N}\mathsf {\_Rep}}(s\,m)) \\ &{}&{}\quad \wedge {\mathsf {OneOne}}\,s \wedge (\forall m {\cdot }\, {\mathsf {Is\_}\mathbb {N}\mathsf {\_Rep}}\,m \Rightarrow \lnot s\,m = z) \\ &{}&{}\quad \wedge \left( \forall p {\cdot }\, p\,z \wedge (\forall m {\cdot }\,p\,m \Rightarrow p(s\, m)) \Rightarrow (\forall m {\cdot }\,{\mathsf {Is\_}\mathbb {N}\mathsf {\_Rep}}\,m \Rightarrow p\,m)\right) \end{array} \end{aligned}$$

The defining property of ${\mathsf {Is\_}\mathbb {N}\mathsf {\_Rep}}$ certainly implies $\exists x {\cdot }\,{\mathsf {Is\_}\mathbb {N}\mathsf {\_Rep}}\,x$ and one may use new_type_definition to introduce a new type $\mathbb {N}$ with defining property:

$$\begin{aligned} \vdash \exists rep :\mathbb {N}\rightarrow {\mathsf {ind}} {\cdot }\,{\mathsf {Type\_Definition}}\,{\mathsf {Is\_}\mathbb {N}\mathsf {\_Rep}}\, rep \end{aligned}$$

From the definition of ${\mathsf {Type\_Definition}}$ and the defining properties of ${\mathsf {Is\_}\mathbb {N}\mathsf {\_Rep}}$ and $\mathbb {N}$, it is then easy to prove the following:

$$\begin{aligned} \begin{array}{lll} \vdash &{}\exists z:\mathbb {N} {\cdot }\,\exists s : \mathbb {N}\rightarrow \mathbb {N} {\cdot }\, \\ &{}\quad {\mathsf {OneOne}}\,s \wedge (\forall m {\cdot }\,\lnot s\,m = z) \wedge \left( \forall p {\cdot }\, p\,z \wedge (\forall m {\cdot }\,p\,m \Rightarrow p(s\, m)) \Rightarrow (\forall m {\cdot }\, p\,m)\right) \end{array} \end{aligned}$$

Applying $\mathtt{new\_specification}$, one introduces constants 0 and ${\mathsf {Suc}}$ whose defining property is as follows:

$$\begin{aligned} \vdash {\mathsf {OneOne}}\,{\mathsf {Suc}}\wedge (\forall m {\cdot }\,\lnot {\mathsf {Suc}}\,m = 0) \wedge (\forall p {\cdot }\, p\,0 \wedge (\forall m {\cdot }\,p\,m \Rightarrow p({\mathsf {Suc}}\, m)) \Rightarrow (\forall m {\cdot }\, p\,m)) \end{aligned}$$

Now standard arguments lead to the principle of definition by recursion^6^:

$$\begin{aligned} \vdash \forall z:\alpha {\cdot }\,\forall r:\alpha \rightarrow \mathbb {N}\rightarrow \alpha {\cdot }\,\exists ! f:\mathbb {N}\rightarrow \alpha {\cdot }\, f\,0 = z \wedge (\forall n {\cdot }\,f({\mathsf {Suc}}\,n) = r\,(f\,n)\,n. \end{aligned}$$

Using this one defines the arithmetic operators and proves all their standard properties. This is a standard pattern in defining a new type: one first uses properties of the representing set to define constructors (0 and ${\mathsf {Suc}}$ here) and then proves a characterising theorem for the constructors (the principle of induction here) from which all subsequent results follow. The precise presentation of the above argument varies from system to system particularly as regards the introduction of constants other than those one expects to find in a theory of arithmetic, e.g., a constant for the representation function $\mathbb {N}\rightarrow {\mathsf {ind}}$. The above account is based on the ProofPower development, in which the only “unexpected” constant is ${\mathsf {Is\_}\mathbb {N}\mathsf {\_Rep}}$ (and that is introduced only to conform with a standardised approach to introducing new types).

The following observation about new_type_definition has been made by several people over the years:

**XX1**: Typically the existence of the representation function is irrelevant once one has proved some abstract characterisation of the new type, e.g., by the existence of constructors satisfying some closure property. It would be more elegant if one could introduce a new type with the abstract characterisation as the defining property.

There are (admittedly somewhat recondite) cases in which the lack of abstractness reported in **XX1** actually results in a real loss of expressiveness. This occurs, for example, in John Harrison’s work on self-verification of HOL Light [7]. To explain this, if *X* and *Y* are sets represented in the usual way as predicates in HOL, let me write $X \preceq Y$ to mean there is a one-to-one mapping of *X* into *Y* and $X \prec Y$ to mean $X \preceq Y \wedge \lnot Y \preceq X$. Harrison needs to introduce a new type $\varUpsilon$ with universe *U*, say, such that for any set *X*, if $X \prec U$, then $\mathbb {P}(X) \prec U$. Now any countably infinite set enjoys this closure property (since powersets of finite sets are finite), so one can define $\varUpsilon$ as a subtype of $\mathbb {N}$. However, that leaves open the possibility that what is subsequently proved depends on $\varUpsilon$ being countable. This is rather unsatisfactory in the context of [7]: Harrison’s script (Model/modelset.ml in the HOL Light distribution) actually allows one to rearrange comments to replace the type definition with an axiom asserting the closure property, giving some evidence that the unwanted information given by the type definition is not actually used.

A solution to **XX1** is actually given in the HOL4 documentation [2], but it has never been implemented. The solution comprises a new principle for defining types that I will call new_type_specification. This takes as input two theorems as follows^7^ :

$$\begin{aligned} \begin{array}{ll} \vdash \exists x:\sigma {\cdot }\, p\,x \\ \vdash (\exists rep :\beta \rightarrow \sigma {\cdot }\, {\mathsf {Type\_Definition}}\,p\, rep ) \Rightarrow q \end{array} \end{aligned}$$

Here *p* is a closed term of type $\sigma \rightarrow {\mathsf {bool}}$ and *q* is a closed term of type ${\mathsf {bool}}$. The type variables in *p* must be contained in a given list $\alpha _1, \ldots , \alpha _n$ of type variables. $\beta$ must be a type variable that is not one of the $\alpha _j$. $\mathtt{new\_type\_specification}$ introduces a new *n*-ary type constructor^8^ ${\mathsf {op}}$ together with the axiom:

$$\begin{aligned} \vdash q[(\alpha _1, \ldots , \alpha _n){\mathsf {op}}/\beta ]. \end{aligned}$$

The idea here is that *q* represents some desired property of the generic instance $(\alpha _1, \ldots , \alpha _n){\mathsf {op}}$ of the new type with $\beta$ standing in for that instance. We prove that property holds on the assumption that there is a one-to-one mapping $rep$ between $\beta$ and the extent of the predicate *p* on the representation type $\sigma$. We then receive the property *q* with $\beta$ instantiated accordingly as the defining property of the new type.

A proof of the model-theoretic conservativness of new_type_specification is given in [2]. The model-theoretic conservativeness of new_type_definition follows from this, since the effect of the latter can be achieved with the former if we take *q* to be $\exists rep :\beta \rightarrow \sigma {\cdot }\, {\mathsf {Type\_Definition}}\,p\, rep$. However, because HOL is not complete with respect to the standard semantics, we cannot deduce from this that new_type_specification is conservative in the proof-theoretic sense. It can be shown that new_type_specification is proof-theoretically conservative using the method of Henkin models [9] (since, $\mathtt{new\_type\_specification}$ is conservative in the model-theoretic sense under the Henkin semantics, but then as the proof system is complete for the Henkin semantics, we can conclude that $\mathtt{new\_type\_specification}$ and hence $\mathtt{new\_type\_definition}$ are proof-theoretically conservative). A direct syntactic proof of the conservativeness of new_type_specification seems much more difficult: an analogous principle for introducing new sorts in many-sorted first-order logic is easily justified using relativisation of quantifiers, but this line of argument does not generalise straightforwardly to the full typed $\lambda$-calculus.

So for example, *p* might be the predicate ${\mathsf {Is\_}\mathbb {N}\mathsf {\_Rep}}$ discussed above and the second theorem might capture the parts of the construction of the natural numbers discussed above that are concerned with the representation type as follows:

$$\begin{aligned} \begin{array}{ll} \vdash &{} (\exists rep :\beta \rightarrow {\mathsf {ind}} {\cdot }\,{\mathsf {Type\_Definition}}\,{\mathsf {Is\_}\mathbb {N}\mathsf {\_Rep}}\, rep ) \Rightarrow {}\\ &{} \quad \begin{array}{ll} &{}(\exists z:\beta {\cdot }\,\exists s:\beta \rightarrow \beta {\cdot }\, {\mathsf {OneOne}}\,s \wedge (\forall m:\beta {\cdot }\,\lnot s\,m = z) \\ &{}\wedge (\forall p {\cdot }\, p\,z \wedge (\forall m {\cdot }\,p\,m \Rightarrow p(s\,m)) \Rightarrow (\forall m {\cdot }\,p\,m))) \end{array} \end{array} \end{aligned}$$

Thus new_type_specification could be used to define the type $\mathbb {N}$ with the defining property asserting the existence of a a zero and a successor function satisfying the principle of induction. The representation type would only appear in the proof of the inputs to new_type_specification.

Used in the context of Harrison’s work on self-verification of HOL Light, new_type_specification would have allowed Harrison to achieve the same effect as achieved with new axiom: the defining property of the new type would comprise only the desired closure properties and would give no upper bound on the cardinality.

Unfortunately, new_type_definition and new_type_specification conflict with the design principle **JH1** requiring the definition of the inference system to be independent of the definition of logical connectives and other defined constructs such as ${\mathsf {Type\_Definition}}$. The existential theorem required as an input to both new_type_definition and new_type_specification is not a big problem: in place of the theorem $\vdash \exists x {\cdot }\,p\,x$ one can ask for a theorem of the form $\vdash p\,w$ where *w* is some term. This reformulation is equivalent to the original, since $\exists x {\cdot }\,p\,x$ is provable iff $p\,(\varepsilon x {\cdot }\,p\,x)$ is. However, the logical connectives appearing inside the definition of ${\mathsf {Type\_Definition}}$ and hence, indirectly, in the output of new_type_definition and in the second part of the input to new_type_specification are much more problematic. HOL Light solves the problem by reformulating new_type_definition so that it not only introduces the new type but also introduces new constants for the abstraction and representation functions. I will borrow the name define_ty_op from OpenTheory for the HOL Light variant of new_type_definition. define_ty_op takes as input a theorem of the form $\vdash p\,w$ where *p* is a closed term of type $\sigma \rightarrow {\mathsf {bool}}$ for some type $\sigma$, and where the type variables occurring in *p* are contained in a list of type variables $\alpha _1, \ldots , \alpha _n$. define_ty_op introduces a new *n*-ary type constructor ${\mathsf {op}}$ and constants $\mathsf {rep}: (\alpha _1, \ldots , \alpha _n){\mathsf {op}}\rightarrow \sigma$ and $\mathsf {abs}: \sigma \rightarrow (\alpha _1, \ldots , \alpha _n){\mathsf {op}}$ satisfying the following axioms:

$$\begin{aligned} \begin{array}{l} \vdash \mathsf {abs}(\mathsf {rep}\,a) = a \\ \vdash p\,r \Leftrightarrow (\mathsf {rep}(\mathsf {abs}\,r) = r) \end{array} \end{aligned}$$

Here, *a* and *r* are free variables of type $(\alpha _1, \ldots , \alpha _n){\mathsf {op}}$ and $\sigma$, respectively, and “$\Leftrightarrow$” is just syntactic sugar for the ${\mathsf {bool}}\rightarrow {\mathsf {bool}}\rightarrow {\mathsf {bool}}$ instance of equality. The first of these axioms implies that $\mathsf {rep}$ is one-to-one and the second implies that the image of $\mathsf {rep}$ is the extent of the predicate *p*, thus together the two axioms imply $\exists rep :(\alpha _1, \ldots , \alpha _n){\mathsf {op}}\rightarrow \sigma {\mathsf {Type\_Definition}}\,p\, rep {\cdot }\,$ (with $\mathsf {rep}$ as the witness). Conversely, from $\exists rep :(\alpha _1, \ldots , \alpha _n){\mathsf {op}}\rightarrow \sigma {\mathsf {Type\_Definition}}\,p\, rep {\cdot }\,$ one can infer the existence of the functions $\mathsf {abs}$ and $\mathsf {rep}$.

Objection **XX1** applies even more strongly for a type definition principle that forces the definition of the representation and abstraction functions as new constants. Once one has an abstract characterisation of a new type, these constants are no longer of any use and so they are potentially misleading clutter (a naive user may be tempted to use them instead of the abstract characterisation).

This raises the problem of finding a way of expressing the abstraction offered by new_type_definition or even better new_type_specification without using the logical connectives. A solution to this problem has recently been found as the eventual outcome of the following objection raised by Mario Carneiro on the implementation of define_ty_op in OpenTheory version 5 (which follows HOL Light in this respect).

**MC1**: In existing implementations of define_ty_op, the names of the free variables *a* and *r* that appear in the axioms it introduces are fixed by the implementation, while in all the other rules the choice of free variable names is determined by the inputs to the rule.

An elegant way to solve **MC1** would be to avoid the use of free variables in the new axioms altogether. A solution along these lines was found by Mario Carneiro himself soon after making the above observation, subject to a slight refinement by Joe Hurd. This solution has been adopted in version 6 of OpenTheory and replaces the two axioms introduced by define_ty_op by the following.

$$\begin{aligned} \begin{array}{l} \vdash (\lambda a {\cdot }\,\mathsf {abs}(\mathsf {rep}\,a)) = (\lambda a {\cdot }\,a) \\ \vdash (\lambda r {\cdot }\,\mathsf {rep}(\mathsf {abs}\,r) = r) = (\lambda r {\cdot }\, p\,r) \end{array} \end{aligned}$$

These are easily seen to be equivalent to the original formulation and as they involve no free variables, this solves the problem of **MC1**. The observant reader will ask why the right-hand side of the second equation is not *p* rather than $\lambda r {\cdot }\,p\,r$: this was Joe Hurd’s refinement: the reason is to separate concerns in the logic by preventing the type definition principle accidentally implying instances of the $\eta$-conversion axiom: given $p = (\lambda r {\cdot }\,\mathsf {rep}(\mathsf {abs}\,r) = r)$, it is an easy exercise in the use of the HOL Light inference system, to derive $p = \lambda x {\cdot }\,p\,x$.

Carneiro and Hurd’s method for capturing the semantics of the representation and abstraction functions also gives a way of expressing the intention of new_type_specification without using the logical connectives. I will call the resulting principle simple_ new_type_specification. We will give the principle in a more general form presently, but to simplify the notation initially we shall first describe a special case. In the special case, the principle takes as input two theorems as follows:

$$\begin{aligned} \begin{array}{c} \vdash p\,w \\ (\lambda a {\cdot }\, abs ( rep \,a)) = (\lambda a {\cdot }\,a), (\lambda r {\cdot }\, rep ( abs \,r) = r) = (\lambda r {\cdot }\,p\,r) \vdash q \end{array} \end{aligned}$$

where (just as in new_type_specification) *p* is a closed term of type $\sigma \rightarrow {\mathsf {bool}}$, *q* is a closed term of type ${\mathsf {bool}}$ and the type variables occurring in *p* are contained in a given list $\alpha _1, \ldots , \alpha _n$ of type variables. $abs$ and $rep$ are free variables of types $\sigma \rightarrow \beta$ and $\beta \rightarrow \sigma$ respectively, $\beta$ being a type variable that is not one of the $\alpha _j$. Just like new_type_specification, simple_new_type_specification introduces a new *n*-ary type constructor ${\mathsf {op}}$ together with the axiom:

$$\begin{aligned} \vdash q[(\alpha _1, \ldots , \alpha _n){\mathsf {op}}/\beta ]. \end{aligned}$$

Note that the free variables $abs$ and $rep$ are in the inputs to the definitional principle and hence do not give rise to objection **MC1**.

As a final remark on the principles for introducing new types, let me note:

**RA3**: Particularly when defining syntax, mutually recursive types are common, but new_type_definition etc. only allow introduction of one type at a time.

simple_new_type_specification as we have described it above deals with objection **XX1** as it stands and as the axioms it introduces contain no free variables it cannot give rise to the the implementation issue of **MC1**. It is easily extended to address the objection **RA3** and this is the general form of the principle that we have promised to give. When used to define *m* types simultaneously, the principle takes as input $m + 1$ theorems as follows (*m* theorems with no assumptions and 1 with 2*m* assumptions):where:

- for $$1 \le i \le m$$:
  - $p_i$ is a closed term of type $\sigma _i \rightarrow {\mathsf {bool}}$;
  - $w_i$ is a term of type $\sigma _i$:
  - the type variables occurring in $p_i$ are contained in $\alpha _{i1}, \ldots , \alpha _{in_i}$;
  - $$ abs _i$$ is a free variable of type $$\sigma _i \rightarrow \beta _i$$;
  - $$ rep _i$$ is a free variable of type $$\beta _i \rightarrow \sigma _i$$;
- $\alpha _{11}, \ldots , \alpha _{1n_1}, \ldots , \alpha _{m1}, \ldots , \alpha _{mn_m}, \beta _1, \ldots , \beta _m$ are distinct type variables;
- *q* is a closed term of type ${\mathsf {bool}}$.

On this input, simple_new_type_specification introduces new *n*-ary type constructors ${\mathsf {op}}_1, \ldots , {\mathsf {op}}_m$ together with the axiom:

$$\begin{aligned} \vdash q[(\alpha _{11}, \ldots , \alpha _{1n_1}){\mathsf {op}}_1/\beta _1, \ldots , (\alpha _{m1}, \ldots , \alpha _{mn_m}){\mathsf {op}}_m/\beta _m]. \end{aligned}$$

That this is model-theoretically conservative may be proved using the methods of the proof for new_type_specification in [2]. That it subsumes the existing mechanisms is clear from the discussion above.

The general form of simple_new_type_specification described above certainly addresses all the technical objections to the existing mechanisms. However, type definitions are much less frequent than constant definitions and are often made using a package, e.g., to define programming language syntax. It is conceivable that the form of simple_new_type_specification that introduces just one new type may be a good compromise between the complexity of the logical principle and its use in practice, given that most users do not interact directly with the underlying definitional principle. An experiment to port one of the existing type definition packages to work with simple_new_type_specification would be a sensible next step in the evaluation of these proposals.

## Concluding Remarks

The reader may have noted that the approach discussed in section 4 will typically introduce a new type whose defining property asserts the existence of various constructor and destructor functions immediately followed by an application of gen_new_specification to introduce constants for those functions. It is certainly possible to give a definitional principle that combines the features of gen_new_specification and simple_new_type_specification, simultaneously introducing new constants and new types (the variables representing abstraction and representation function would be permitted in the witnesses for the new constants). Details are left to the reader. The resulting definitional principle would subsume all the others considered in this paper. However it is not clear whether the extra complexity of such a rule merits the relatively modest reduction in clutter.

It is noteworthy that with a little care both the inference rules and the axiomatization of the logical connectives in HOL can be given in a form that is intuitionistically acceptable [8]. The law of the excluded middle only enters as a consequence of the axiom of choice when presented using Hilbert’s choice operator [6]. Freek Wiedijk has made the interesting remark that the forms of type definition that provide an abstraction function as well as the representation function are not intuitionistically acceptable: new_type_definition is intuitionistically acceptable, but define_ty_op is not. To see the problem with define_ty_op, let $\phi$ be any closed term of type ${\mathsf {prop}}$ in higher-order intuionistic propositional calculus. (Here we write ${\mathsf {prop}}$ rather than ${\mathsf {bool}}$ for the type of propositions to avoid a classical bias). E.g $\phi$ might be the sentence $\forall p {\cdot }\, p \vee \lnot p$. Now consider the function $\chi = \lambda p {\cdot }\, p \vee \lnot p \wedge \phi$ of type ${\mathsf {prop}}\rightarrow {\mathsf {prop}}$. Then $\chi (\top )$ holds and so, with define_ty_op, we can construct a new type, $\tau$ say, in one-to-one correspondence with the extent of $\chi$. If we have abstraction and representation functions, $\mathsf {abs}: {\mathsf {prop}}\rightarrow \tau$ and $\mathsf {rep}: \tau \rightarrow {\mathsf {prop}}$, the composite $f = \mathsf {rep}\circ \mathsf {abs}: {\mathsf {prop}}\rightarrow {\mathsf {prop}}$ then satisfies:

$$\begin{aligned} \begin{array}{ll} {(i)} &{} \forall p {\cdot }\, f(f(p)) = f(p) \\ { (ii)} &{} \forall p {\cdot }\, f(p) = p \Leftrightarrow p \vee \lnot p \wedge \phi \end{array} \end{aligned}$$

Now let $q = f(\bot )$. By *(i)*, $f(q) = q$, whence by *(ii)*, we have $q \vee \lnot q \wedge \phi$, and so $q \vee \lnot q$ holds. Now, by *(ii)*, $q = \bot$ iff $\bot \vee \lnot \bot \wedge \phi$, which is equivalent to $\phi$. So, if we assume *q*, then $q \not = \bot$ and we may deduce $\lnot \phi$, while if we assume $\lnot q$, then we are assuming $q \Rightarrow \bot$, whence $q = \bot$, and we may deduce $\phi$. Hence we have $q \vee \lnot q \Rightarrow \lnot \phi \vee \phi$, but as we have already observed $q \vee \lnot q$ holds, and hence so does $\lnot \phi \vee \phi$. Thus a type definition principle providing the abstraction function will enable us to prove $\lnot \phi \vee \phi$ for any closed sentence $\phi$ and this is not intuitionistically acceptable. This raises the apparently difficult challenge of formulating an intuitionistically acceptable type definition principle that avoids expanding out the definitions of the logical connectives in the definition of ${\mathsf {Type\_Definition}}$ 9 . However, few people deliberately restrict themselves to the purely intutionistic fragment of HOL, so this is a question of mainly philosophical interest.

This paper has been concerned with definitions in higher-order logic, but definitional principles like $\mathtt{new\_specification}$ are also of interest in implementations of first-order logic. ACL2’s encapsulate command [12] has much in common with $\mathtt{new\_specification}$. From an historical perspecive, it is interesting that the development of the precursor of encapsulate, the CONSTRAIN facility in NQTHM [5], was contemporary with the introduction of $\mathtt{new\_specification}$ into HOL.

To return to higher-order logic, our new principle of constant definition seens to have been accepted by HOL developers and we believe it will prove a useful tool in improving the quality of specifications in HOL. The problem of reconciling the differences in the facilities offered for type definition between the various HOL implementations via a common abstraction of what they provide that meets the design desiderata of all the systems is a harder one, but we believe the work reported in this paper has made some useful progress in that direction.

## Footnotes

1 The details of the mechanism for specifying the names of new constants are not important for present purposes.

2 At various places in this note, I sketch observations made by other people. The wording used is mine and not theirs and any misrepresentation is my responsibility.

3 One of the referees made the interesting suggestion that one could get rid of the existential quantifier by expanding it using the definition given in [8]. However, one would also have to expand out the universal quantifiers, conjunctions and implications on which that definition depends. The resulting term:

$$\begin{aligned}&\lambda p {\cdot }\, \left( \lambda P {\cdot }\, P = \left( \lambda x {\cdot }\, (\lambda p {\cdot }\, p) = (\lambda p {\cdot }\, p)\right) \right) \left( \lambda q {\cdot }\, \left( \lambda p {\cdot }\, \lambda q {\cdot }\, \left( \lambda p {\cdot }\, \lambda q {\cdot }\, \left( \lambda f {\cdot }\, f\,p\,q\right) = \left( \lambda f {\cdot }\, f \left( (\lambda p {\cdot }\, p) \right. \right. \right. \right. \right. \\&\left. \left. \left. \left. \left. \quad = (\lambda p {\cdot }\, p)\right) \left( (\lambda p {\cdot }\, p) = (\lambda p {\cdot }\, p)\right) \right) \right) p\,q = p\right) \left( \left( \lambda P {\cdot }\, P = \left( \lambda x {\cdot }\, (\lambda p {\cdot }\, p)\right. \right. \right. \right. \\&\left. \left. \left. \left. \quad = (\lambda p {\cdot }\, p)\right) \right) \left( x \left( \lambda p {\cdot }\, \lambda q {\cdot }\, \left( \lambda p {\cdot }\, \lambda q {\cdot }\, (\lambda f {\cdot }\, f\,p\,q)\right. \right. \right. \right. \right. \\&\left. \left. \left. \left. \left. \quad = \left( \lambda f {\cdot }\, f \left( (\lambda p {\cdot }\, p) = (\lambda p {\cdot }\, p)\right) \left( (\lambda p {\cdot }\, p) = (\lambda p {\cdot }\, p)\right) \right) \right) p\,q = p\right) (p\,x) q\right) \right) q\right) \end{aligned}$$

contains 118 subterms: 32 variable occurrences, 12 occurrences of the equality constant, 43 applications and 31 $\lambda$-abstractions. To include such a monster in the definition of the logic seems far worse than accepting a one-off definitional principle for bootstrapping. Moreover, an implementation would be required to include in its logical kernel some trustworthy means for recognising instances of this term.

4 In ProofPower, certain performance-critical aspects of the implementation of the semantic embedding of Z [18] have been fine-tuned around new_specification and an opportunity to rework this code has not yet arisen. In HOL4, some significant refactoring would be required to avoid a use of new_specification prior to the point in the system build where new_specification is defined in terms of gen_new_specification and the best way to go about that refactoring is still under discussion.

5 The means for specifying the name of the new type constructor ${\mathsf {op}}$ and the list $\alpha _1, \ldots , \alpha _n$ are not important here. ProofPower is slightly more general than HOL4 in allowing $\alpha _j$ that do not appear in $\sigma$ or *p*. However, the extra generality appears to be of no practical importance.

6 Note that with gen_new_specification, we could now take this as the definition of 0 and ${\mathsf {Suc}}$ if we wished, as suggested in observation **RA2**.

7 The principle as described in [2] allows both theorems to have additional closed formulas in the assumptions, but this does not add any extra generality, so we prefer the simpler form.

8 The means for specifying the name of the new type constructor ${\mathsf {op}}$ and the list of type variables $\beta , \alpha _1, \ldots , \alpha _n$ are not important here.

9 Freek Wiedijk has proposed three axioms for the representation function that are intuitionistically acceptable and are classically equivalent to the axioms returned by define_ty_op. These axioms are $\mathsf {rep}\,a = \mathsf {rep}\,b \vdash a = b$ (giving that $\mathsf {rep}$ is one-to-one), $\vdash p(\mathsf {rep}\,a)$ (giving that the range of $\mathsf {rep}$ is contained in the extent of *p*) and $p\,r, (\lambda a {\cdot }\, r = \mathsf {rep}\,a) = (\lambda a {\cdot }\, t) \vdash t$ (implying, taking $t = \bot$, that the range of $\mathsf {rep}$ contains the extent of *p* in classical logic). Unfortunately, in intutionistic logic, these axioms do not seem to imply the result $\vdash \forall r {\cdot }\, p\,r \Rightarrow (\exists a {\cdot }\, r = \mathsf {rep}\,a)$ that one would wish to hold when defining a new type in the intuitionistic fragment of HOL.
